# Supplementary material for: Phosphoinositide 3-Kinase P110δ-Signaling Is Critical for Microbiota-Activated IL-10 Production by B Cells that Regulate Intestinal Inflammation
Source: Cells. 2019 Sep 21;8(10):1121. doi: 10.3390/cells8101121 (PMC6829312; doi:10.3390/cells8101121)
Supplement: Supplementary file 1 [file cells-08-01121-s001.pdf]

Table S1. Antibodies used in the present study.

Antibody	Clone number	Conjugated	Brand
anti-mouse B220	RA3-6B2	Pacific Blue	Invitrogen
anti-mouse CD11b	M1/70	PE-Cy7	BD Pharmingen
anti-mouse CD11c	HL3	Alexa Fluor 700	BD Pharmingen
anti-mouse CD16/CD32	2.4G2	-	BD Pharmingen
anti-mouse CD19	6D5	Brilliant Violet 605	BioLegend
anti-mouse CD1d	1B1	PE	eBioscience
anti-mouse CD21	7G6	Brilliant Violet 650	BD Pharmingen
anti-mouse CD23	B3B4	PE	eBioscience
anti-mouse CD24	M1/69	APC	BD Pharmingen
anti-mouse TCR-β	H57-597	Alexa Fluor 700	BD Pharmingen
anti-mouse CD3e	145-2C11	PE-Cy5	eBioscience
anti-mouse CD4	RM4-5	APC	BioLegend
anti-mouse CD45	30-F11	Pacific Orange	Invitrogen
anti-mouse CD5	53-7.3	PE-Cy5	BD Pharmingen
anti-mouse CD8a	53-6.7	PE-Cy7	eBioscience
anti-mouse FOXP3	FJK-16s	PE	eBioscience
anti-mouse IgD	11-26c.2a	APC	BD Pharmingen
anti-mouse IgM	II/41	PE-Cy7	eBioscience
anti-mouse MHC-II	M5/114.15.2	Brilliant Violet 605	BioLegend
anti-mouse CD64	X54-5/7.1	PE	BioLegend
anti-mouse Ly6G	1A8	APC	BioLegend
anti-mouse NK-1.1	PK136	Brilliant Violet 650	BD Pharmingen
LIVE/DEAD Fixable Dead Cell Stain Kit	N/A	Near-IR	Molecular Probes
mouse IgG1 κ	MOPC-21	PE	BioLegend
mouse IgG2a κ	G155-178	Brilliant Violet 605	BD Pharmingen
rat IgG2a κ	R35-95	APC, PE-Cy5, PE-Cy7	BD Pharmingen
rat IgG2a κ	RTK2758	Brilliant Violet 605, Pacific Blue, APC	BioLegend
rat IgG2a κ	eBR2a	PE, PE-Cy7	eBioscience
rat IgG2b κ	A95-1	PE-Cy7, APC, Pacific Orange	BD Pharmingen
rat IgG2b κ	R35-38	Brilliant Violet 650, PE,	BD Pharmingen
hamster IgG1 λ2	A19-3	Alexa Fluor 700	BD Pharmingen
hamster IgG2 λ1	Ha4/8	Alexa Fluor 700	BD Pharmingen
hamster IgG	eBio299Am	PE-Cy5	eBioscience
anti-mouse CD3e	R35-95	-	R&D systems
anti-mouse CD28	794716	-	R&D systems
anti-mouse CD210 (IL-10R)	1B1.3a	-	BD Pharmingen
rat IgG1 κ	R3-34	-	BD Pharmingen
mouse IgG1 κ	MOPC-21	-	BioLegend

Figure S1

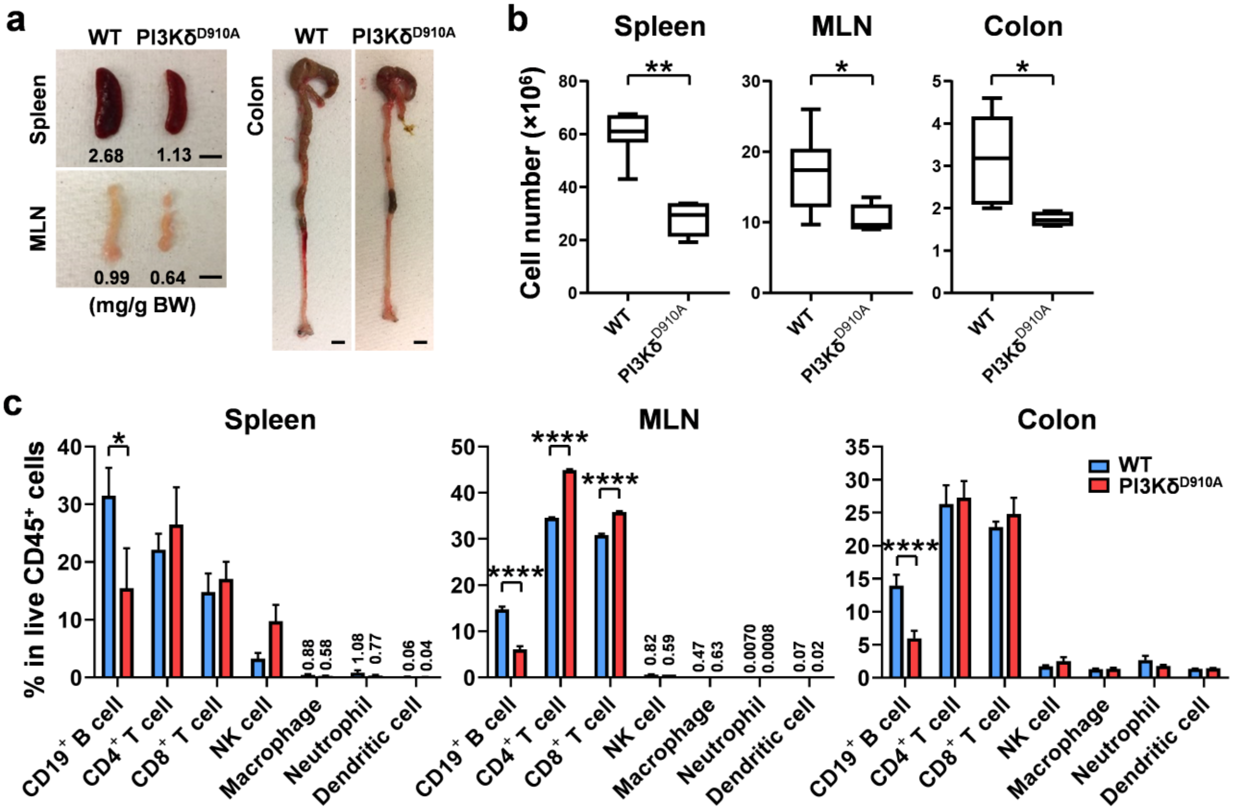

Figure S1. B cell population is significantly reduced in PI3Kδ^{D910A} mice. (**a**) Representative spleen, mesenteric lymph node (MLN), and colon tissues from 8-10 week-age male WT and PI3Kδ^{D910A} mice. Bar indicates 0.5 cm. Number indicates tissue weight (mg) per body weight (g). (**b**) Cell numbers of spleen cells, MLN cells, and colonic lamina propria (cLP) cells of 8-10 week-age WT and PI3Kδ^{D910A} mice. Mean ± range. N=4-5/group. (**c**) The percentages of B cells (B220$^+$CD19$^+$), CD4$^+$ T cells (TCRβ$^+$CD3$^+$CD4$^+$CD8^{neg}), CD8$^+$ T cells (TCRβ$^+$CD3$^+$CD4^{neg}CD8$^+$), natural killer (NK) cells (TCRβ^{neg}NK1.1$^+$), macrophages (TCRβ^{neg}CD11b$^+$CD64$^+$), neutrophils (TCRβ^{neg}MHCII^{neg}Ly6G$^+$) and dendritic cells (TCRβ^{neg}CD64^{neg}MHCII$^+$CD11c$^+$) in singlet live CD45$^+$ spleen cells, MLN cells or cLP cells of 8-10 week-age WT or PI3Kδ^{D910A} mice. Blue bar (left) and red bar (right) indicate WT and PI3Kδ^{D910A} respectively. Mean ± SEM. N=4-5/group. Mann-Whitney unpaired two-tailed test was used. *$p < 0.05$, **$p < 0.01$, ****$p < 0.0001$.

Figure S2

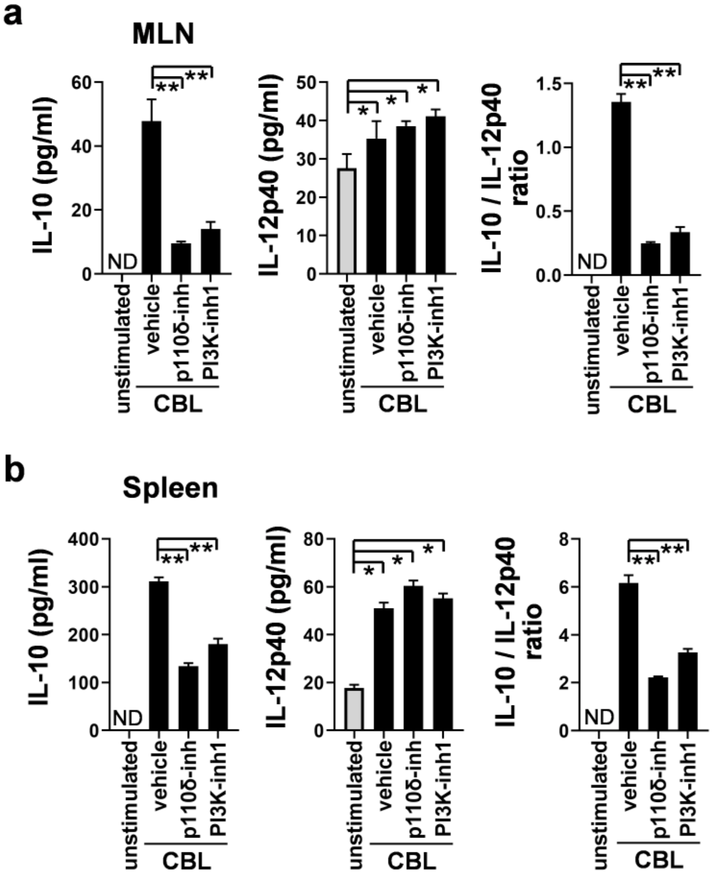

Figure S2. The inflammatory or regulatory phenotypes of MLN and spleen cells are determined by PI3Kδ signaling. (**a**, **b**) 1×10^6 unfractionated MLN cells (**a**), or spleen cells (**b**) from WT mice were cultured with or without CBL (10 μg/ml) in the absence or presence of several PI3K-related inhibitors in complete medium in 96-well plates for 24 hours. Supernatant levels of IL-10 and IL-12p40 were measured by ELISA. PI3K-related inhibitors included: PI3Kp110δ-selective inhibitor (p110δ-inh, IC87114, 2 μM) and PI3K-global inhibitor (PI3K-inh1: LY294002, 2 μM). Vehicle control: dimethyl sulfoxide. N=4-5/group. Mean ± SEM. Dunn's multiple comparisons test following one-way ANOVA was used. *$p < 0.05$, **$p < 0.01$. ND indicates not detected.

Figure S3

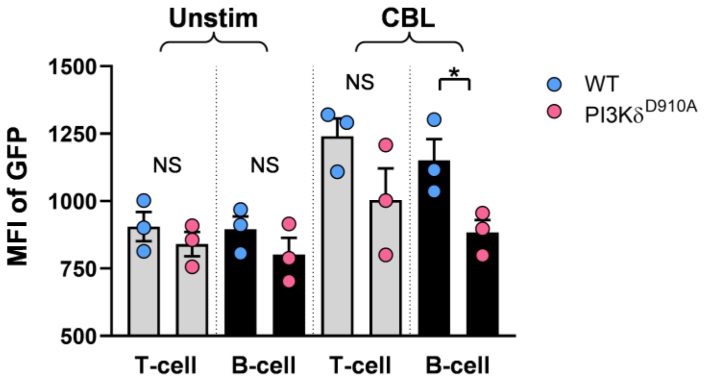

Figure S3. The median fluorescence intensities of GFP in T cells and B cells. 1×10^6 unfractionated MLN cells from 8-10 week-age WT; *Il10*^{eGFP} or PI3Kδ^{D910A} mice were cultured with medium alone (unstim) or CBL (10 μg/ml) in complete medium for 24 hours at 37°C with 5% CO_2. The percentages of GFP$^+$ T cells (CD45$^+$TCRβ$^+$CD3$^+$) and B cells (CD45$^+$B220$^+$CD19$^+$) in MLN cells (CD45$^+$) stimulated with CBL are shown. The median fluorescence intensities (MFI) of GFP in GFP$^+$ T cells and B cells in MLN cells (CD45$^+$) stimulated with CBL are shown. Mean ± SEM. Mann-Whitney unpaired one-tailed test was used. *$p < 0.05$. NS indicates not significant.

Figure S4

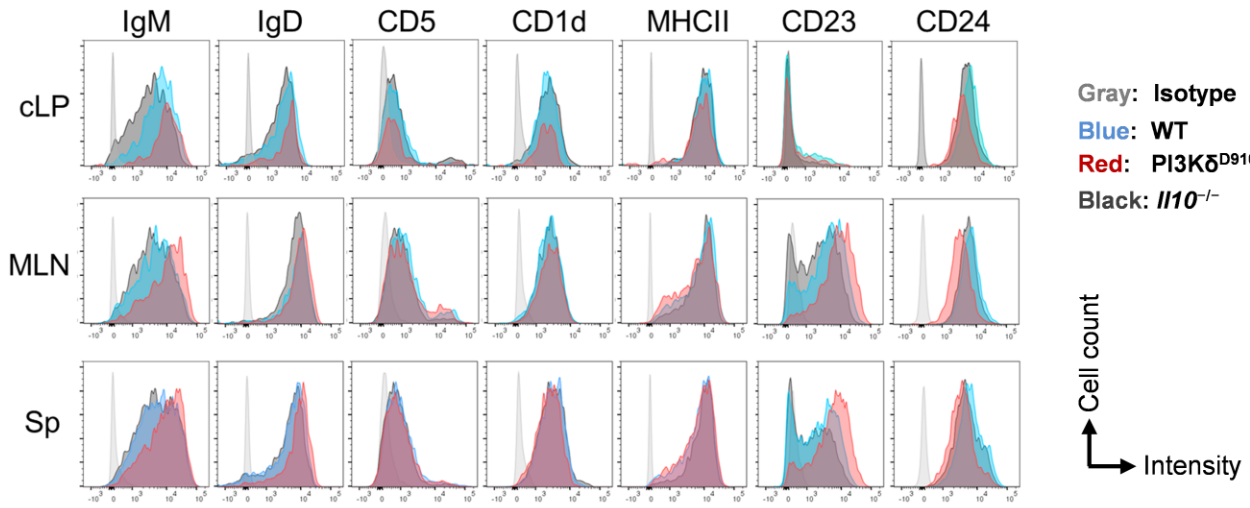

Figure S4. Phenotypic characteristics of B cells in PI3Kδ^{D910A} mice. Colonic lamina propria (cLP), mesenteric lymph node (MLN), or spleen (Sp) cells were isolated from 8-10 week-age normal PI3Kδ (WT); *Il10*^{eGFP}, PI3Kδ^{D910A}; *Il10*^{eGFP}, *Il10*$^{-/-}$ mice. The singlet live B cell populations (Live/Dead^{neg}CD45^{+}B220^{+}CD19^{+}) were analyzed by flow cytometry with the following phenotypic markers: IgM (PE-Cy7), IgD (APC), CD5 (PE-Cy5), CD1d (PE), MHC-II (Brilliant Violet 650), CD23 (PE), and CD24 (APC). Representative histograms of B cells are shown. Gray: isotype control, Blue: WT, Red: PI3Kδ^{D910A}, Black: *Il10*$^{-/-}$.

Figure S5

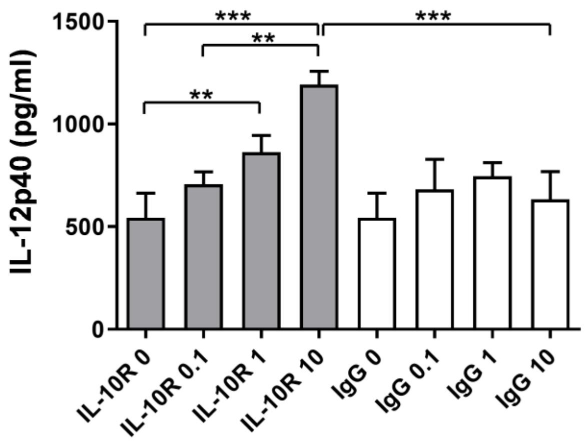

Figure S5. Blockade of IL-10 signaling increases IL-12p40 secretion by B cells. Unfractionated WT splenic B cells were cultured in the presence of indicated concentrations (0-10 ng/ml) of anti-IL-10 receptor antibody (IL-10R) or isotype control antibody (IgG) with 500 nM CpG-DNA stimulation for 72 hours. IL-12p40 supernatant level was determined by ELISA. Mean ± SEM, triplicates. N=4-5/group. Dunn's multiple comparisons test following one-way ANOVA was used. ** $p < 0.01$, *** $p < 0.001$.
